# Anæmia

**Published:** 1888-02

**Authors:** 


ANÆMIA. By Frederick P. Henry, M. D. Re-printed from Polyclinic. Philadelphia: P. Blakiston, Son & Co., 1887.

This little treatise is the first in English on this subject. It is well written and deals systematically with the topics under discussion. An interesting point noted under the anæmia of typhoid fever is that in this disease the blood is normal in respect to number of red corpuscles, but deficient in fluidity. The therapeutical deduction is to supply fever patients with water. Many persons suffer, even while able to go about, from lack of water. Babies are almost universally tortured in this way and suffer from deprivation of simple water to quench thirst. The book deals very satisfactorily and thoroughly with the subject of anæmia in its various forms. H. H.

